# In Vitro Effects of *Streptomyces* tyrosinase on the Egg and Adult Worm of *Toxocara vitulorum*

**Published:** 2020

**Authors:** Hatem SHALABY, Heba ASHRY, Moataza SAAD, Tarek FARAG

**Affiliations:** 1. Department of Parasitology and Animal Diseases, National Research Center, Giza, Egypt; 2. Department of Microbial Chemistry, National Research Center, Giza, Egypt

**Keywords:** *Streptomyces*, Tyrosinase, Protease, *Toxocara vitulorum*, In-vitro, Bio-control

## Abstract

**Background:**

Several species of streptomycetes, saprophytic bacteria found widely distributed in soil, water and plants, produce bioactive compounds such as intra and extracellular hydrolases including lytic enzymes which reflecting on their importance in the biological control of insects and parasites. This study assessed the in vitro effects of *Streptomyces* tyrosinase, produced from *Streptomyces* spp. isolated from Egyptian soil, on animal-parasitic nematode *Toxocara vitulorum*, in terms of egg development and adult worm’s cuticular structure, and as an alternative strategy to alleviate this infection.

**Methods:**

This study was conducted at the National Research Centre, Egypt in 2018. Five different concentrations of tyrosinase, ranged from 1%–30% were tested against the development of *T. vitulorum* eggs. The concentration induced the highest inhibitory activity was tested against adult *T. vitulorum* cuticle, which is essential for the protective and nutritive functions. The results were compared with those observed in the egg development and worm cuticle following incubation in *Streptomyces* protease (as a reference enzyme).

**Results:**

Compared to *Streptomyces* protease, higher inhibitory activity on *T. vitulorum* egg development and extreme cuticular alterations of the treated adult worms had been observed following 24 h exposure to *Streptomyces* tyrosinase. Once the cuticle had been damaged, the enzyme would be able to penetrate deeper into the internal tissues of the nematode and caused more widespread disruption.

**Conclusion:**

The current study could offer a promising bio-control agent, *Streptomyces* tyrosinase, against *T. vitulorum* alternative to the more expensive synthetic anthelmintics.

## Introduction

The actinomycetes like streptomycetes are originally thought of to be an intermediate cluster between bacteria and fungi and living free, saprophytic bacteria found cosmopolitan in soil, water, and plants. Many species of streptomycetes produce bioactive compounds such as intra and extracellular hydrolases including lytic enzymes that reflective of their importance in the biological control of insects and parasites (1–3). One of the enzymes is protease suggested as the central enzyme in the lysis of pathogenic bacterial and fungal cell wall, acting on the peptide bands formed by specific amino acids to hydrolyze them (4). Proteases were used as insecticidal agents (5).

The *Fasciola gigantica* ovicidal effect of the protein concentrations of extracellular lytic enzymes produced by *Streptomyces griseolus* increased with increasing the protein concentration and also the protease activity (6). Another lytic enzyme is tyrosinase, which is a type 3 copper-containing enzyme that can react with exposed tyrosyl side chains in polypeptides (7). It has many medicinal and industrial applications. For example, it has commercial importance in the production of L-3, 4-dihydroxyphenylalanine (L-DOPA), one of the preferred drugs for the treatment of Parkinson‘s disease, and due to its ability to react with phenols, it can be applied in detoxification of phenol-containing wastewater and contaminated soils (8). Recently, tyrosinase was found to be a potent anticancer agent (9).

On the other hand, helminth infections are the foremost common parasitic infections of animals worldwide. They have constrained animal productivity, both in developing and developed countries (10). *Toxocara vitulorum* helminth is one of the most important parasites of buffaloes, particularly buffalo calves of one to three months of age, which inhabits the small intestine of these animals and causes severe damage to the intestinal mucous membrane (11). Besides 8000 to 100,000 eggs per gram faeces are shed by *T. vitulorum* adult female per day. A thick eggshell provides protection against adverse environmental conditions like chemical and physical harm, permitting eggs to stay alive for several years (12). It is of zoonotic importance, humans become infected by inadvertent ingestion of eggs containing third larval stage (13), which may distribute over the body, causing visceral larva migrans and ocular larva migrans (14).

The control of this helminthic infection still relies heavily on the use of anthelmintics. At a time, in many developing countries people cannot afford to buy anthelmintic drugs. One of the reasons is that these drugs are costly and sometimes unavailable to smallholder farmers. Therefore, due to the cost of animal treatment, besides emergence of anthelmintic resistance, the use of alternative strategy to alleviate this infection should be one of the main attentions such as biological control.

The present study was carried out in vitro investigations: (a) evaluate the ovicidal effect of *Streptomyces* tyrosinase on embryonation of *T. vitulorum* eggs, and (b) examine its morphological effects on adult *T. vitulorum* cuticle, which is essential for the protective and nutritive functions. The results of tyrosinase were compared with those observed in the egg development and the worm cuticle structure following incubation in *Streptomyces* protease that showed, in a previous study, high inhibitory activity on the development of the *Fasciola gigantica* immature eggs (6).

## Materials and Methods

This study was conducted in 2018 at the National Research Centre, Egypt.

### Organisms

*Streptomyces ghanesis* SAH1_CWMSG and *Streptomyces pseudogrisiolus* NRC-15 were previously isolated, identified and used to produce tyrosinase and protease (9, 15).

### Tyrosinase production and Growth conditions

*Streptomyces ghanesis* SAH1_CWMSG was chosen as the most potent tyrosinase producer from our previous studies (9). The production medium was a medium contained per liter: Starch, 1%, Casein hydrolysate 10g/L, K _2_ HPO _4_ 0.5g/L, MgSO _4_ 0.25g/L, L- tyrosine 1.0 g/L. and potassium phosphate buffer to a final concentration of 0.05 M, pH 7.0. Fifty ml of liquid media was dispensed into each 250 ml Erlenmeyer flask and autoclaved at 121 °C for 20 min. The flasks were inoculated in duplicates with 3×10 ^−6^ of the vegetative cells from a seven-day-old *Streptomyces* sp. SAH1_CWMSG. The inoculated flasks were kept at 28±2 °C on a rotary shaker (New Brunswick Scientific Co., NJ, USA) at 150 rpm for 6 d. The uninoculated fermentation medium was used as a negative control during the experiment. The contents of each flask were harvested by centrifugation at 8000 rpm for 10 min and the supernatant was analyzed for determined enzyme activity and cell growth (9).

### Protease production and Growth conditions

The inoculum was prepared by growing the organism on soybean slants for 9 d; then, 1ml of spore suspension containing 3.5 × 10 ^−7^ was made by using sterile saline solution. One ml of spore suspension was inoculated on 49 ml liquid medium, which contained (g/L) 20, sucrose; 0.5, NaCl; 2, KNO _3_ ; 1, K _2_ HPO _4_ ; 0.5, MgSO _4_ ; 3, CaCO _3_ and 0.01 for each of FeSO _4_ , ZnSO _4_ and MnCl _2_ in 250 ml Erlenmyer flasks. Thereafter, the flasks were incubated for 6 d at 30 °C in a shaking incubator (200 rpm). The culture medium was centrifuged at 6000 rpm for 10 min and the supernatant was separated as protease enzyme (15).

### Protein concentration

The protein content of the tested enzyme was determined before its storage in small vials at − 80 °C (16).

### In vitro ovicidal effect of tyrosinase and protease on T. vitulorum eggs

The eggs were obtained from the adult female worms after incubation in Ringer solution for 3 h at 37 °C in an atmosphere of 5% CO_2_, and processed according to the method of Oshima (17). Number of unembryonated eggs per 1 ml of solution was determined and kept in the refrigerator at 4 °C until used. For assessment of the inhibitory activity of tyrosinase and protease enzymes on development of unembryonated eggs, suitable medium-sized Petri dishes, each contained 5000 eggs in five different concentrations of each tested enzyme, 1%, 5%, 10%, 20% and 30%, in doublets at 24 h exposure time were prepared. Untreated eggs were incubated as a normal control group. At the end of the exposure period, several times of washing, and sedimentation using distilled water were carried out to get rid of the remnant of the tested enzymes. The rate of embryonic development in both exposed and control eggs was evaluated (17). The reduction percentage of *T. vitulorum* egg development induced by the tested enzymes was estimated using the following formula:

$$\text{Inhibitory}\;\text{activity}=\frac{\%\;\text{of}\;\text{controleggs}\;\text{containingL}3-\%\;\text{of}\;\text{exposedeggs}\;\text{containingL}3}{\%\;\;\text{controleggs}\;\text{containingL}3\;}\times 100$$

### In vitro effect of tyrosinase and protease on the cuticle of T. vitulorum adult worms

Adult worms of *T*. *vitulorum* were collected from the intestines of naturally infected buffalo calves slaughtered in a local abattoir in Cairo province, Egypt. After recovery, the worms were washed and transferred to Ringer solution (18), containing the tested enzyme at 30% concentration. The whole worms were incubated for 24 h at 37°C in an atmosphere of 5% CO _2_ . Another group was prepared by incubating worms in Ringer solution without enzyme addition, as normal control worms. Six worms were examined for each group.

### Light microscopy

Following incubation, the adult worms were cut into small, 5 mm pieces before being fixed in 10% buffered formol saline, dehydrated, cleared and embedded in paraffin blocks. Paraffin sections of 5 μ thickness were prepared and stained by Haematoxylin and Eosin (H&E) according to the method of Bancroft et al. (19). The cuticle of adult worms was examined and photographed using an Olympus CX41 microscope.

## Results

### In vitro effect of tyrosinase and protease on development of T. vitulorum eggs

**Fig. 1:**
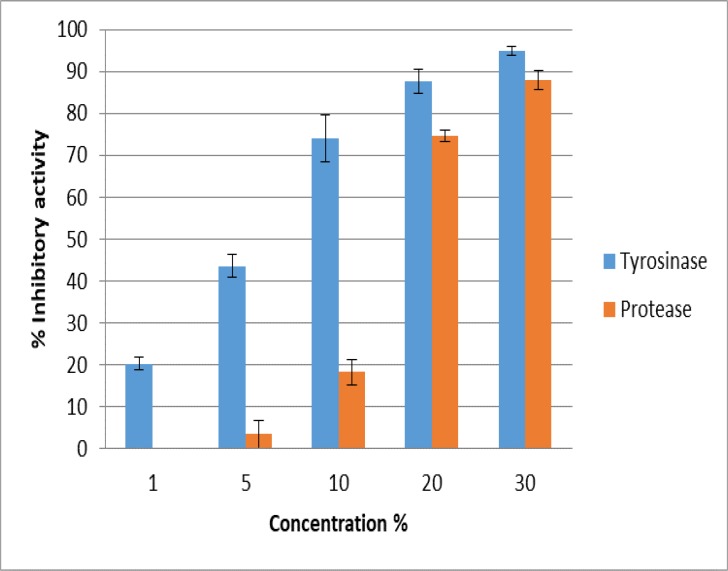
Inhibitory activity of tyrosinase and protease on development of *T. vitulorum* eggs

Five different concentrations of tyrosinase and protease ranged from 1%–30% were tested against the development of *T. vitulorum* eggs. Most of *T. vitulorum* eggs remained in developmental stages, with absence of L3 larvae development, after exposure to 30% concentration of both tested enzymes. Yet, tyrosinase induced higher inhibitory activity than that observed with protease at all tested concentrations (Fig. 1).

**Fig. 2:**
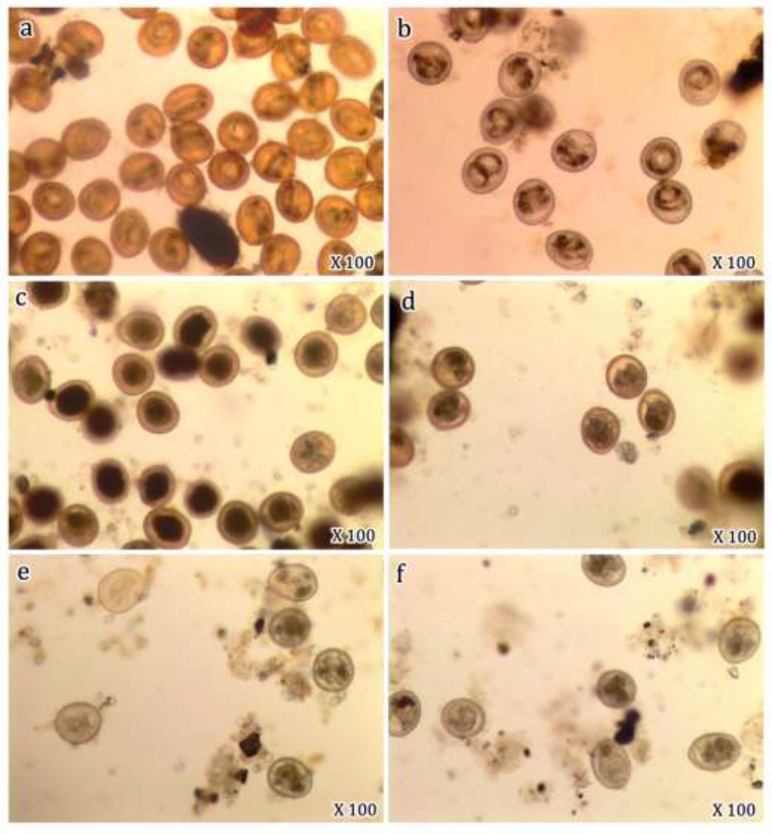
*T. vitulorum* eggs. **(a, b)** Embryonated control eggs contained third larval stage. **(c, d)** After exposure to 30% concentration of tyrosinase enzyme. Note the majority of eggs’ development is arrested at one-cell stage. **(e, f)** After exposure to 30% concentration of protease enzyme. Note irregular cell division in developed eggs

The egg development was arrested at one-cell stage, and if development occurred, the irregular cell division was observed resulting in atypical blastomeres (Fig. 2). The inhibitory activity of tyrosinase was concentration dependent. A slight inhibitory effect of tyrosinase; 20.3% and 43.6% was observed at 1% and 5% concentration, respectively. This inhibitory effect increased with increasing application concentration, till reaching the highest rate (94.9%) at concentration of 30%. While following exposure to protease, no detectable inhibitory activity on egg development was recorded in 1 and 5% concentrations. A slight inhibitory effect of protease (18.3%) was observed at 10% concentration and increased with increasing application concentration till reaching 88.0% at 30% concentration (Fig. 1).

### In vitro effect of tyrosinase and protease on the cuticle of T. vitulorum adult worms Control worms

**Fig. 3:**
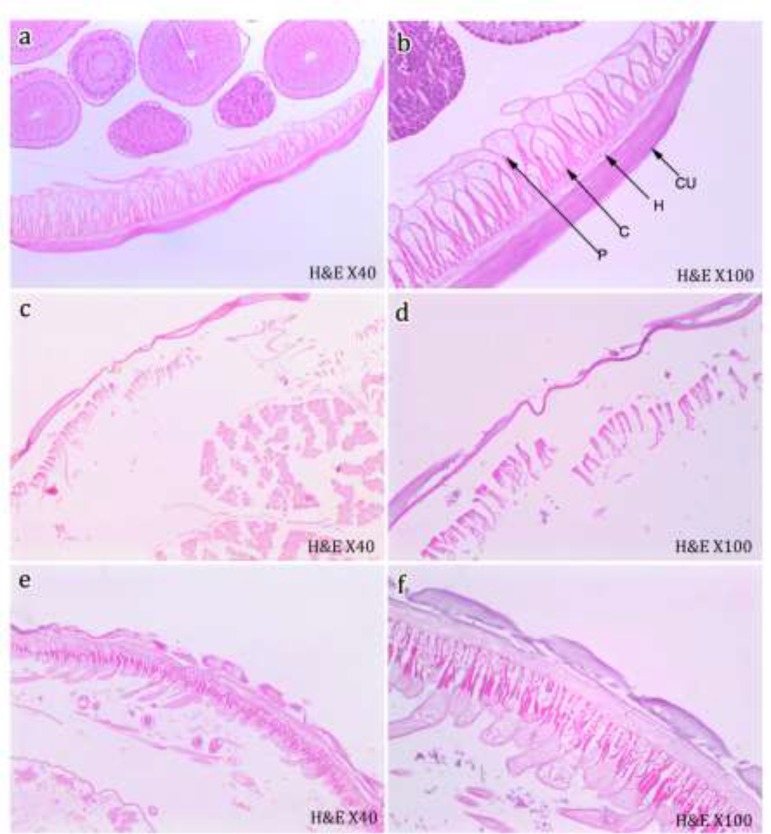
Light micrographs (LMs) of the cuticle cross-section of adult *T. vitulorum*. **(a, b)** Normal control worms. **(c, d)** Following 24 h incubation with 30% concentration of tyrosinase enzyme. Note extreme disruption of the cuticle and the underlying muscle cells. **(e, f)** Following 24 h incubation with 30% concentration of protease enzyme. LMs show areas of disconnection between the cuticle and the hypodermis as well as severe vacuolization of the fibril contractile muscular portion. CU, cuticle; H, hypodermis; C, contractile part of the muscle cells; P, cytoplasmic part of the muscle cells

The cuticle morphology of control worms; incubated in medium without enzyme addition, appeared normal when examined via light microscopy. A brief description of some of the important cuticular features was necessary to assess the changes resulting from tyrosinase and protease treatment. The adult worm was covered by a sort of dense non-cellular cuticle consisting of different layers and overlying a thin hypodermis. Connected of the last might have been an extensive number of longitudinally arranged, striated muscle cells. Those muscle cells required a fibril contractile muscular portion directed toward hypodermis and a granular non-contractile cytoplasmic portion projecting toward the center of the body (Fig. 3a, b).

### Treated worms

Following 24 h incubation with tyrosinase at 30% concentration, the treated worms showed extreme disruption of the outer cuticular layer and hypodermis, as well as severely folded and wrinkled inner cuticular layer. Moreover, the muscle underlying the cuticle exhibited complete disruption (Fig. 3c, d). While less cuticular alterations were observed in the adult worms; following 24 h incubation with protease at 30% concentration. Where large areas of disconnection between the cuticle and the hypodermis could be observed and the former took the form of a corrugated layer. The muscle cells were deformed and there appeared several large vacuoles in the fibril contractile muscular portion (Fig. 3e, f)

## Discussion

Numerous microorganisms can attack and destroy nematodes by varying ways, such as trapping, parasitizing, and producing toxins and enzymes. Extracellular lytic enzymes appear to be potent factors that can deteriorate the essential chemical components of the nematode eggshell and cuticle (20).The nematode eggshell contains chitin fibrils embedded in a protein matrix (21), and the nematode cuticle mainly consists of proteins, including collagens (22).

In this study, the in vitro effects of *Streptomyces* tyrosinase on animal-parasitic nematode *T. vitulorum*, in terms of egg development and cuticular structure were assessed. As far as we know, it is the first study evaluating *Streptomyces* tyrosinase for the development of new bio-control agent against the animal-parasitic nematodes. At a time, numerous extracellular enzymes capable of digesting the main corresponding chemical constituents of the plant-parasitic nematode cuticle and eggshell (protein, chitin, and lipids) had been isolated and identified in various nematophagous fungi and bacteria (3, 23–26). In this study, *Streptomyces* protease showed high inhibitory activity on *T. vitulorum* egg development and induced severe cuticular alterations in the treated adult worms. In this sense, serine proteases had been reported as pathogenic factors found in bacterial or fungal pathogens against insects, nematodes, and vertebrates (27, 28). *Streptomyces* protease had shown ovicidal activity against animal-parasitic trematode *F. gigantica* eggs (6). Moreover, the bioinsecticides produced by actinomycete isolates were active against the housefly, *Musca domestica*, and induced up to 90% larval mortality (2). Overall, 100% mortality of the 3 ^rd^ instar larvae of *Culex quinquefasciatus* (mosquito) and *Tribolium castaneum* (Red flour beetle) were recorded by using the metabolites of actinomycetes (29). The fungi *Duddingtonia flagrans* and *Monacrosporium sinense* had shown a lytic effect on eggs of *Ascris lumbricoides* (30). Furthermore, *Artobotrys robusta* and *A. conoides* had the same effect against *Toxocara cains* eggs (31).

Interestingly, compared to *Streptomyces* protease, higher inhibitory activity on *T. vitulorum* egg development and extreme cuticular alterations of the treated adult worms had been observed following exposure to *Streptomyces* tyrosinase. The nematode cuticle is almost completely composed of protein, and sixteen amino acids have been detected in the hydrolyzed cuticles of the biologically related nematodes; *Ascaris lumbricoides* and *T. mystax*, including tyrosine (32).

The only enzymes which are known to attack simple, intact proteins in vitro are proteolytic ones. Tyrosinase catalyzes the oxidation by oxygen of many phenolic derivatives such as tyrosine. It is a bifunctional enzyme that catalyzes two types of reactions: the *ortho-*hydroxylation of monophenols to its corresponding *o*-diphenol and the oxidation of diphenols to its correspondent *ortho*-quinones (33). Therefore, tyrosinase was involved in the detoxification of phenolic compounds (34, 35). It was also suggested to be a potent tool in treating melanoma (36).

In this study, disorganization of the cuticle and body musculature was observed in adult worms exposed to *Streptomyces* tyrosinase. Once the cuticle had been damaged, the enzyme would be able to penetrate deeper into the internal tissues of the nematode and caused more widespread disruption, as shown in the light micrographs. The cuticular distortion and degenerative changes in the subcuticular region were common features of drug-treated parasites and had been described for several nematode species exposed to anthelmintics in vitro as previously summarized (37). This damage would certainly obstruct some of the important functions of the nematodes’ body wall such as nutrition and immunoprotection (38).

## Conclusion

The current study could offer a promising bio-control agent, *Streptomyces* tyrosinase, against *T. vitulorum* alternative to the more expensive synthetic anthelmintics. It showed a clear inhibitory effect on *T. vitulorum* egg development and had a destructive effect on the cuticle of adult worm in vitro. Consequently, it might help to reduce the occurrence of infective eggs in the host environment after treatment. However, its in-vivo nematocidal efficiency needs to be accessed.
